# Effects of early pelvic floor muscle rehabilitation combined with continuity of care on pelvic floor electromyography and postpartum depression in women with postpartum uterine prolapse

**DOI:** 10.3389/fgwh.2026.1837604

**Published:** 2026-06-19

**Authors:** Wu Ling, Xiaomei Peng, Yuan Li, Linxia Chen, Hongshen Pan, Haiyan Yang, Weihua Zhong

**Affiliations:** Department of Obstetrics and Gynecology, The Fifth People's Hospital of Chengdu, Chengdu, Sichuan, China

**Keywords:** continuity of care, pelvic floor electromyography, pelvic floor muscle training, postpartum depression, postpartum uterine prolapse

## Abstract

**Objective:**

This study aimed to evaluate the effects of early pelvic floor muscle rehabilitation training combined with continuity of care on pelvic floor electromyography (EMG) and postpartum depression in women with postpartum uterine prolapse.

**Methods:**

A total of 100 women with postpartum uterine prolapse were enrolled and randomly assigned to an intervention group or a control group (*n* = 50 per group) using a random number table. Outcome assessors were blinded to group allocation to minimize assessment bias. The control group received early pelvic floor muscle rehabilitation training alone, while the intervention group received additional continuity of care. At baseline, small but statistically significant differences in Self-Rating Anxiety Scale and Self-Rating Depression Scale scores were observed between the groups; to account for this, baseline values were included as covariates in the statistical adjustment.

**Results:**

After the intervention, pelvic floor muscle strength was significantly better in the intervention group than in the control group (*P* < 0.05), and the proportion of women with muscle strength grade ≥3 was significantly higher in the intervention group (*P* < 0.05). No significant between-group differences in pelvic floor EMG parameters were observed at baseline (*P* > 0.05). At 1 and 3 months post-intervention, both pre-resting and post-resting mean EMG values decreased significantly from baseline in both groups (*P* < 0.05), with significantly lower values in the intervention group than in the control group (*P* < 0.05). After adjusting for baseline differences, both anxiety and depression scores were significantly reduced in both groups at 3 months post-intervention (*P* < 0.05), and the reductions were significantly greater in the intervention group (*P* < 0.05).

**Conclusion:**

In this preliminary study, early pelvic floor muscle rehabilitation training combined with continuity of care was associated with improvements in pelvic floor EMG parameters and reduced postpartum depressive symptoms in women with postpartum uterine prolapse. Larger multicenter trials are needed to confirm these findings.

## 1 Introduction

Pelvic floor dysfunction (PFD) is a common disorder in women, encompassing a range of conditions such as urinary and fecal incontinence, uterine prolapse, pelvic organ displacement, and chronic pelvic pain (1). Pregnancy and childbirth are major risk factors for PFD. Previous studies have shown that more than 60% of women experience some degree of PFD after pregnancy and delivery, which can significantly impair quality of life (2). Among these disorders, uterine prolapse is a severe form of PFD and is associated with adverse psychological outcomes, including postpartum anxiety and depression, further diminishing wellbeing. With growing awareness of health and quality of life, an increasing number of women recognize the importance of pelvic floor rehabilitation. Although some women initiate pelvic floor muscle training early after delivery, the therapeutic effect is often limited due to inappropriate techniques, lack of professional guidance, or poor adherence.

Recent randomized controlled studies have further highlighted the value of technology-assisted rehabilitation in postpartum recovery. For example, Afzal et al. (3) conducted a randomized controlled trial (RCT) investigating postpartum diastasis recti, which evaluated surface electromyography (EMG) biofeedback-assisted core strengthening exercises compared with conventional non-assisted exercise. This work parallels the present study in two key ways: first, it utilized surface EMG as an objective electrophysiological tool to assess musculoskeletal rehabilitation outcomes, mirroring our use of pelvic floor EMG to evaluate pelvic floor function; second, its study design compared assisted, guided exercise intervention vs. standard rehabilitation alone, which mirrors our comparison of rehabilitation with additional continuity of care vs. standard rehabilitation. The findings of the previous study support the use of objective EMG indicators for rehabilitation assessment and suggest that enhanced, guided intervention strategies may improve adherence and functional recovery. Similar to these approaches, the present study adopted pelvic floor electromyography as an objective assessment tool and explored whether continuity of care could further optimize rehabilitation outcomes (3). Therefore, this study aimed to evaluate the effects of early pelvic floor muscle rehabilitation combined with continuity of care on pelvic floor EMG parameters and postpartum depression in women with postpartum uterine prolapse.

## 2 Materials and methods

### 2.1 Study design and participants

This was a single-center RCT. A total of 100 women diagnosed with postpartum uterine prolapse between March 2024 and March 2025 were enrolled in this study. Participants were randomly assigned to an intervention group or a control group (*n* = 50 per group) using a random number table. All participants delivered at the obstetrics department of our hospital and received postpartum rehabilitation interventions at the same institution.

Sample size calculation was performed based on the primary outcome, the pre-resting pelvic floor EMG value at 3 months post-intervention. Based on a preliminary pilot study, we assumed a between-group difference of 0.65 μV in the pre-resting EMG value, with a standard deviation of 0.9 μV. With a two-sided *α* level of 0.05 and a power of 80%, the required sample size was 43 participants per group. To account for a potential 15% loss to follow-up, we enrolled 50 participants per group, resulting in a total sample size of 100 participants.

To minimize assessment bias, outcome assessors (including the staff performing EMG assessment, muscle strength grading, and scale administration) were blinded to group assignment. Due to the nature of the behavioral intervention, participants and intervention providers could not be blinded to group allocation.

The study was approved by the Ethics Committee of The Fifth People's Hospital of Chengdu, and written informed consent was obtained from all participants and their families.

### 2.2 Inclusion and exclusion criteria

#### 2.2.1 Inclusion criteria

Participants were eligible if they

1. were diagnosed with uterine prolapse at our hospital and
2. had a singleton pregnancy with cephalic presentation.

#### 2.2.2 Exclusion criteria

Participants were excluded if they

1. had severe respiratory or circulatory system disease,
2. were unable to complete the assessment scales,
3. were unable to continue functional exercise after discharge due to external factors (e.g., lower limb injury),
4. had concomitant neurological disease, or
5. had a history of abdominal surgery.

### 2.3 Interventions

#### 2.3.1 Control group

Participants in the control group received early pelvic floor muscle rehabilitation training. Before discharge, the severity of uterine prolapse and pelvic floor muscle strength were assessed, and an individualized rehabilitation program was developed. Initial training intensity was set at a moderate level to enhance adherence, and the exercise plan was gradually intensified as patients adapted. Structured health education was provided by senior attending physicians, covering postpartum hygiene, wound care, pelvic floor self-care, risk factors for pelvic floor injury, pelvic floor anatomy and function, exercise techniques, and self-assessment of muscle strength.

Pelvic floor muscle training was conducted by rehabilitation staff. Participants were instructed to contract and elevate the urethra, perineum, and anus as much as possible, hold the contraction for at least 3 s, and then relax slowly. After a 5–8 s rest, the maneuver was repeated. Each session lasted 15–30 min and was performed two to three times daily. Participants were advised to maintain regular daily routines and avoid activities that increase intra-abdominal pressure (e.g., prolonged sitting, squatting, or heavy lifting). At discharge, each participant received a rehabilitation booklet with instructions on self-care and precautions (e.g., no tub bathing or sexual intercourse for 2 months postpartum). Both groups received the same standard follow-up reminder contacts to ensure attendance at scheduled assessment visits, including telephone reminders 3 days before each assessment.

#### 2.3.2 Intervention group

In addition to the intervention received by the control group, participants in the intervention group received additional continuity of care, primarily delivered via WeChat-based health education and follow-up.

- WeChat group and public account: Patients followed the department's WeChat public account and joined a postpartum uterine prolapse rehabilitation group for information and peer support.
- Peer communication: Patients were encouraged to share experiences and correct misunderstandings within the group.
- Online education and Q&A: Regular online sessions were held, and targeted follow-up was conducted via WeChat and telephone. Some patients attended outpatient visits for program adjustment when necessary. Follow-up focused on prolapse severity, recovery of pelvic floor muscle strength, and progress in exercises.
- Psychological intervention: For patients experiencing anxiety or depression (often related to uterine prolapse), underlying causes were explored, and coping strategies were discussed. Education about uterine prolapse was provided to reduce uncertainty and fear, along with active rehabilitation guidance and psychological support to strengthen confidence and alleviate negative emotions.

#### 2.3.3 Follow-up management

To minimize loss to follow-up, multiple strategies were implemented. All participants received a follow-up schedule at discharge. The intervention group was additionally engaged via a WeChat group, with weekly reminders and personalized messages. Both groups received telephone reminders 3 days before each scheduled assessment (1 and 3 months postpartum). If a participant was unreachable by phone, a text message or WeChat message was sent, and up to three additional contact attempts were made within 1 week. If a participant missed a scheduled visit, an outpatient appointment was rescheduled within the following week. Through these efforts, no participant was lost to follow-up during the 3-month study period.

### 2.4 Outcome measures

The primary outcome was pelvic floor EMG (pre-resting mean value at 3 months). Secondary outcomes included pelvic floor muscle strength, Self-Rating Anxiety Scale (SAS) score, and Self-Rating Depression Scale (SDS) score. This trial was not registered in a clinical trial registry; future trials should be prospectively registered. The Consolidated Standards of Reporting Trials (CONSORT) checklist was consulted in preparing this report.

The following outcomes were assessed:

1. Pelvic floor muscle strength at 3 months post-intervention.
2. Pelvic floor EMG parameters before intervention, at 1 month, and at 3 months post-intervention.
3. Anxiety and depression scores (SAS and SDS) before intervention and at 3 months post-intervention.

#### 2.4.1 Pelvic floor muscle strength

Pelvic floor muscle strength was evaluated using the Modified Oxford Scale (4), ranging from grade 0 (no contraction) to grade 5 (strong contraction).

#### 2.4.2 Pelvic floor electromyography

Pelvic floor EMG was assessed using the Glazer method (5). The pre-resting and post-resting mean values were used as the primary EMG indicators.

#### 2.4.3 Anxiety and depression

Anxiety and depression were evaluated using the SAS and SDS (6). These are widely used general screening tools for psychological distress; however, it should be noted that they are not validated specifically for the postpartum population. The Edinburgh Postnatal Depression Scale (EPDS) is the standard validated instrument for postpartum depression research, which would have been more appropriate for this study population, and this represents a limitation of the present study. All 100 distributed questionnaires were returned.

### 2.5 Statistical analysis

All statistical analyses followed the intention-to-treat (ITT) principle, where all randomized participants were included in the analysis regardless of protocol adherence. As all participants completed the 3-month follow-up with no missing data, no additional missing data handling was required.

Statistical analysis was performed using R version 4.3.1 (R Foundation for Statistical Computing, Vienna, Austria). A mixed model for repeated measures (MMRM) was used to analyze longitudinal outcomes with repeated measurements, which accounts for the correlation within participants over time. Critically, the model included baseline values of each outcome as covariates, to adjust for any baseline imbalance between groups, including the observed baseline differences in psychological scores. The model also included fixed effects for group, time point, and the group-by-time interaction.

Normally distributed continuous data are presented as least squares (LS) means with 95% confidence intervals (CIs), unless otherwise specified. Between-group differences are presented as the difference in LS means with 95% CIs, adjusted for baseline values. Categorical variables are presented as counts and percentages, and were compared using the chi-square test or Fisher's exact test as appropriate. A two-sided *P-*value <0.05 was considered statistically significant.

## 3 Results

### 3.1 Participant flow

A total of 100 participants were enrolled and randomized, with 50 assigned to the intervention group and 50 to the control group. All participants completed the 3-month follow-up assessment, resulting in a 100% follow-up rate. No participants were excluded from the analysis.

### 3.2 Baseline characteristics

The baseline characteristics of the participants are presented in Table 1. No significant differences were found between the two groups in age, body mass index (BMI), educational level, mode of delivery, neonatal birth weight, or severity of uterine prolapse at baseline (all *P* > 0.05), indicating that the randomization successfully balanced the baseline characteristics between the two groups for these variables. However, small but statistically significant differences were observed in the baseline psychological scores, which were adjusted for in the statistical analysis.

**Table 1:** Baseline characteristics of the two groups.

Variable	Intervention group (*n* = 50)	Control group (*n* = 50)	*P*
Age (years, mean ± SD)	24.31 ± 4.21	24.45 ± 4.13	>0.05
BMI (kg/m^2^, mean ± SD)	25.65 ± 2.35	25.71 ± 2.43	>0.05
Education level, n (%)			
Primary/junior middle school	12 (24.0)	11 (22.0)	>0.05
Senior high school	17 (34.0)	19 (38.0)	
College or above	21 (42.0)	20 (40.0)	
Mode of delivery, *n* (%)			
Vaginal delivery	23 (46.0)	24 (48.0)	>0.05
Cesarean section	27 (54.0)	26 (52.0)	
Neonatal birth weight (g, mean ± SD)			
Male infant	3,136 ± 186	3,127 ± 198	>0.05
Female infant	3,121 ± 165	3,145 ± 158	>0.05
Degree of uterine prolapse, *n* (%)			>0.05
Grade I	12 (24.0)	13 (26.0)	
Grade II	32 (64.0)	30 (60.0)	
Grade III	6 (12.0)	7 (14.0)	

### 3.3 Pelvic floor muscle strength

After intervention, pelvic floor muscle strength was significantly better in the intervention group than in the control group (*P* < 0.05). The proportion of women with muscle strength grade ≥3 was also significantly higher in the intervention group (68.0% vs. 30.0%, *P* < 0.001; Table 2).

**Table 2:** Comparison of pelvic floor muscle strength between the two groups after intervention.

Group	Grade 1, *n* (%)	Grade 2, *n* (%)	Grade 3, *n* (%)	Grade 4, *n* (%)	Grade 5, *n* (%)	Grade ≥3, *n* (%)
Intervention (*n* = 50)	8 (16.0)	8 (16.0)	15 (30.0)	12 (24.0)	7 (14.0)	34 (68.0)
Control (*n* = 50)	16 (32.0)	19 (38.0)	8 (16.0)	5 (10.0)	2 (4.0)	15 (30.0)
*P*-value			0.010			<0.001

Data are presented as *n* (%). Grade ≥3 indicates muscle strength of grade 3 or above.

### 3.4 Pelvic floor electromyography

No significant differences in pelvic floor EMG parameters were observed between the two groups at baseline (*P* > 0.05). At both 1 and 3 months post-intervention, the pre-resting and post-resting mean EMG values decreased significantly from baseline in both groups (*P* < 0.05). At 1 month, the intervention group had significantly lower pre-resting (LS mean difference: −0.52, 95% CI: −0.78 to −0.26, *P* < 0.001) and post-resting (LS mean difference: −0.72, 95% CI: −1.01 to −0.43, *P* < 0.001) EMG values compared to the control group. At 3 months, the differences remained significant: the pre-resting LS mean difference was −0.65 (95% CI: −0.92 to −0.38, *P* < 0.001) and the post-resting LS mean difference was −1.21 (95% CI: −1.52 to −0.90, *P* < 0.001; Table 3).

**Table 3:** Comparison of pelvic floor electromyography between the two groups.

Parameter/group	Before intervention, LS mean (95% CI)	One month after the intervention, LS mean (95% CI)	Three months after the intervention, LS mean (95% CI)
Pre-resting mean value (μV)			
Intervention group (*n* = 50)	5.31 (5.12 to 5.50)	4.41 (4.22 to 4.60)	3.71 (3.52 to 3.90)
Control group (*n* = 50)	5.33 (5.14 to 5.52)	4.93 (4.74 to 5.12)	4.36 (4.17 to 4.55)
Between-group difference (95% CI)	−0.02 (−0.29 to 0.25)	−0.52 (−0.78 to −0.26)	−0.65 (−0.92 to −0.38)
*P*-value	0.887	<0.001	<0.001
Post-resting mean value (μV)			
Intervention group (*n* = 50)	5.92 (5.73 to 6.11)	4.21 (4.02 to 4.40)	3.15 (2.96 to 3.34)
Control group (*n* = 50)	5.95 (5.76 to 6.14)	4.93 (4.74 to 5.12)	4.36 (4.17 to 4.55)
Between-group difference (95% CI)	−0.03 (−0.30 to 0.24)	−0.72 (−1.01 to −0.43)	−1.21 (−1.52 to −0.90)
*P*-value	0.824	<0.001	<0.001

### 3.5 Anxiety and depression scores

At baseline, the between-group differences in SAS and SDS scores were statistically significant (*P* = 0.045 and *P* = 0.047, respectively). To account for this baseline imbalance, we included the baseline values of the psychological outcomes as covariates in our statistical models to adjust for the pre-existing differences between groups, ensuring that the post-intervention comparisons were not confounded by baseline differences.

After adjustment, both SAS and SDS scores decreased significantly from baseline in both groups at 3 months post-intervention (*P* < 0.05). The intervention group had significantly lower adjusted SAS scores (LS mean difference: −13.91, 95% CI: −16.23 to −11.59, *P* < 0.001) and adjusted SDS scores (LS mean difference: −8.02, 95% CI: −10.56 to −5.48, *P* < 0.001) compared to the control group at 3 months (Table 4).

**Table 4:** Comparison of SAS and SDS scores between the two groups.

Outcome/group	Before intervention, mean (SD)	Three months after intervention, adjusted LS mean (95% CI)
SAS score		
Intervention (*n* = 50)	60.24 ± 3.35	43.24 (41.35 to 45.13)
Control (*n* = 50)	59.83 ± 3.32	57.15 (55.26 to 59.04)
Between-group difference (95% CI)	0.41 (−2.28 to 3.10)	−13.91 (−16.23 to −11.59)
*P*-value	0.045	<0.001
SDS score		
Intervention (*n* = 50)	66.21 ± 3.26	50.33 (48.44 to 52.22)
Control (*n* = 50)	68.22 ± 3.25	58.35 (56.46 to 60.24)
Between-group difference (95% CI)	−2.01 (−4.70 to 0.68)	−8.02 (−10.56 to −5.48)
*P*-value	0.047	<0.001

SAS, Self-Rating Anxiety Scale; SDS, Self-Rating Depression Scale. Baseline differences in psychological scores were adjusted for in the statistical models to account for pre-intervention imbalance.

## 4 Discussion

Uterine prolapse is the descent of the uterus from its normal anatomical position along the vaginal canal, often accompanied by prolapse of the anterior or posterior vaginal wall (7). Because the vaginal walls are adjacent to the bladder and rectum, uterine prolapse may coexist with cystocele, urethrocele, or rectocele (8, 9). Previous studies have shown that uterine prolapse is an independent risk factor for postpartum depression, and the incidence of postpartum depression is significantly higher in women with uterine prolapse than in those without (10, 11). The relationship between pelvic floor dysfunction and psychological distress may be explained through multiple mechanisms. From a physiological perspective, persistent pelvic discomfort, functional limitation, and altered pelvic support may directly impair daily activities and quality of life. From a psychosocial perspective, concerns regarding body image, sexual function, and uncertainty about recovery may increase emotional vulnerability during the postpartum period.

Similar associations between structural musculoskeletal disorders and adverse mental health outcomes have also been reported in other clinical settings, suggesting that chronic functional impairment may contribute to psychological burden through shared biopsychosocial pathways. For example, a recent 10-year nationwide cohort study conducted in South Korea found that adults with scoliosis, a chronic structural spinal condition, had a significantly higher risk of subsequent psychiatric morbidity compared to the general population (12). This conceptually parallel finding supports the broader hypothesis that chronic structural and functional physical impairment can lead to long-term psychological distress, regardless of the specific anatomical site of the condition, which helps contextualize the observed relationship between uterine prolapse and postpartum depression in the present study. Therefore, improving pelvic floor function may indirectly alleviate depressive symptoms by restoring physical confidence and reducing illness-related stress, and postpartum pelvic floor rehabilitation may contribute to the prevention and management of postpartum depression.

Pelvic floor EMG is used to evaluate sphincter dysfunction. The striated muscles of the pelvic floor differ from other skeletal muscles in that they contain a higher proportion of type I fibers, particularly in the external sphincter and puborectalis muscles (13). These muscles maintain tonic contraction even during sleep, and they relax during defecation. Thus, EMG can assess pelvic floor muscle function and neural innervation by recording electrical activity at rest and during contraction. Abnormal EMG findings have been associated with pelvic floor disorders such as urinary incontinence and uterine prolapse (14). In this study, we focused on pre-resting and post-resting mean EMG values as key indicators. Our results showed that after intervention, pelvic floor muscle strength and the proportion of women with strength grade ≥3 were significantly higher in the intervention group than in the control group. Both pre-resting and post-resting mean EMG values decreased significantly after intervention in both groups, with greater reductions in the intervention group. These findings suggest that early pelvic floor muscle rehabilitation combined with continuity of care effectively improves pelvic floor muscle function in women with uterine prolapse. Forster et al. (15) similarly reported that continuity of care improved pelvic floor EMG outcomes in postpartum women, likely due to sustained rehabilitation, improved adherence, the timely correction of techniques, and individualized program adjustments.

Regarding psychological outcomes, after adjusting for baseline differences, both scores decreased significantly in both groups, with greater improvements in the intervention group. This indicates that the combined intervention effectively reduces postpartum depression in women with uterine prolapse. However, it should be noted that psychological outcomes were assessed using SAS and SDS, which are general screening instruments rather than postpartum-specific assessment tools. Future studies should incorporate validated postpartum depression scales, such as the EPDS, to improve assessment specificity. Hagen et al. (16) reported similar findings. The benefit may be related to improved health awareness, increased opportunities for guided training, enhanced motivation, and expanded post-discharge follow-up, allowing timely adjustment of intervention strategies.

This study has several limitations. First, it was a single-center study with a relatively small sample size, which may limit the generalizability of the findings. Second, the follow-up period was limited to 3 months, preventing assessment of long-term outcomes. Third, the intervention included multiple components, including online education, psychological support, and individualized follow-up, making it difficult to isolate the independent contribution of each component.

Fourth, the control group received routine discharge education and written rehabilitation guidance, with only standard follow-up reminder contacts, whereas the intervention group received more frequent post-discharge contact and follow-up, including WeChat-based interactions, online education, and regular check-ins. The observed group differences may therefore reflect nothing more than attention bias—the intervention group simply received more clinician contact and engagement, regardless of the specific content of the continuity of care intervention. The possibility of attention bias cannot be completely excluded, and part of the observed benefit may be related to increased clinical engagement rather than the specific content of continuity care itself. Future studies should include attention-matched control conditions, where the control group receives the same frequency of clinician contact without the specific continuity of care intervention, to better clarify the independent effect of continuity care.

In addition, although SAS and SDS are widely used for psychological screening, they may be less specific than the EPDS for identifying postpartum depressive symptoms. Future multicenter studies with larger samples and longer follow-up are warranted.

## 5 Conclusion

In this sample of 100 women with postpartum uterine prolapse, early pelvic floor muscle rehabilitation training combined with continuity of care was associated with beneficial effects on pelvic floor EMG parameters and postpartum depression, after adjusting for baseline imbalance. These preliminary findings suggest that this combined approach may have potential clinical value for postpartum rehabilitation practice. However, due to the single-center design, limited sample size, and potential attention bias, larger multicenter randomized controlled trials with longer follow-up periods and attention-matched control groups are needed to confirm these results and evaluate the long-term efficacy of this intervention.

## Data Availability

The original contributions presented in the study are included in the article/Supplementary Material, further inquiries can be directed to the corresponding author.
